# In vivo demonstration of a novel non-invasive model for inducing localized hypothermia to ameliorate hepatotoxicity

**DOI:** 10.1038/s41598-021-98078-6

**Published:** 2021-09-20

**Authors:** Yeong Lan Tan, Min En Nga, Han Kiat Ho

**Affiliations:** 1 Department of Pharmacy, Faculty of Science, National University of Singapore, Singapore, 117543 Singapore; 2 NUS Graduate School for Integrative Sciences & Engineering, Centre for Life Sciences, National University of Singapore, Singapore, 119077 Singapore; 3 Department of Pathology, National University Hospital, Singapore, 119074 Singapore

**Keywords:** Medical research, Pathogenesis, Health care, Therapeutics

## Abstract

Moderate hypothermia (32 °C) has been previously shown to ameliorate drug-induced liver injuries in vitro. However, there are concerns regarding its clinical relevance as it remains a challenge to perform selective liver cooling in a non-invasive manner. To reconcile this dilemma, we propose the use of pulsed cooling for regional hypothermic conditioning in liver. This involves intermittent cooling applied in pulses of 15 min each, with a one-hour recovery interval between pulses. Cooling is achieved by applying ice packs to the cutaneous region overlying the liver. Through an in vivo C57BL/6NTac mouse study, we demonstrated the feasibility of attaining localized hypothermia close to the liver while maintaining core body temperature. This has successfully ameliorated acetaminophen-induced liver injury based on the liver function tests, liver histology and total weight change. Collectively, we provide a proof of concept for pulsed external localized cooling as being clinically actionable to perform induced selective hypothermia.

## Introduction

We have previously reported on the potential of using moderate hypothermia (32 °C) to ameliorate acetaminophen-induced liver injury (AILI) in vitro^1^ and presented various mechanistic insights on the effect of hypothermia in AILI^2^. Notwithstanding its demonstrated efficacy, the conduct of systemic hypothermia in clinical settings drew skepticism as this could incur non-specific adverse effects such as a slowdown in overall metabolism^3^. While local hypothermia may evade these unfavorable outcomes, the current practice of targeted liver cooling involves an invasive in situ procedure, which may not justify its use in the management of hepatotoxicity. Briefly, it involves the delivery of a cooling solution through the portal vein into the liver with anterograde drainage via the vena cava or hepatic veins^4^. Such a procedure may not offer a prompt and convenient option for timely management of acute drug-induced liver injury (DILI) which has the propensity to worsen drastically within a short timeframe. Therefore, to accentuate the role of hypothermic therapy in managing hepatotoxicity, the key consideration would involve the design of a non-invasive cooling modality that is suitable and closely targeted to specific visceral sites.

For that, we explored the practice of chronic intermittent cold (CIC) exposure and innovated a pulsed cooling approach to achieve regional hypothermic conditioning with minimal invasiveness. By convention, CIC exposure was often performed to evaluate the effects of cold stress in animal models. It may entail severe systemic hypothermia at 4 °C, conditioned for variable intermittent periods of 3–6 h/day for up to six weeks^5,6^. Rather than a continuous cooling, CIC exposure could circumvent prolonged physiological perturbations which may necessitate regular monitoring. Furthermore, Wang et al*.* reported an upregulation of cold shock protein (CSP) expression in the liver following CIC exposure^6^, where CSP was previously shown to play an imperative role in conferring cytoprotection^1^. Collectively, all these reports present intermittent cooling as a viable approach for achieving therapeutic hypothermia in the toxic liver. Yet, prolonged extreme cooling employed in CIC exposure could evoke several undesirable consequences - besides increasing oxidative stress^6^, it may also enhance sympathetic activity^5^. A direct implementation of CIC exposure beyond animal models in a clinical setting is therefore impractical. Hence, we have attempted to improvise the concept of CIC by applying three primary changes. Firstly, moderate hypothermic conditioning would be performed concurrently with drug-induced liver injury (DILI), since liver preservative effects were evident in the past study when hepatocytes were conditioned with moderate hypothermia^1^. This averts potential destructive effects associated with severe hypothermia (< 30 °C). Secondly, the duration of hypothermia would be reduced to shorter pulse of 15-min periods applied hourly for four hours, rather than sustained periods, based on the known pathology of most acute DILI in which the onset of toxicity may occur rapidly within 24 h of exposure. We arrived at the empirical interval and duration of pulsed cooling based on an in vitro study which demonstrated hepatoprotection with three cycles of 26 °C hypothermia, conditioned for ten minutes each^7^. Thirdly, instead of systemic hypothermia, regional surface cooling would be performed cutaneously over the region of the liver. By doing so, we attempt to increase the proximity between the liver and the site of cold application to improve the cooling efficiency in a non-invasive manner.

With this model of a novel, non-invasive pulsed cooling modality, we sought to validate its feasibility and efficacy for the amelioration of acute DILI through an in vivo mouse study. Acetaminophen (APAP) was used as the model toxicant in accordance with past studies of hypothermic conditioning in AILI in vitro^1,2^. Herein, the study first involved a pilot phase to evaluate the feasibility of attaining regional cooling close to the liver. Thereafter, we investigated the effectiveness of pulsed cooling in combating AILI in a larger sample size in the core study. Finally, a series of mechanistic study were performed to characterize the hypothermic behavior of pulsed cooling. Together, this preliminary study outlines the pursuit for a practical, non-invasive cooling strategy targeted at a specific visceral location.

## Results

### Regional cooling close to the liver is feasible with a pulsed cooling procedure

**Figure 1:**
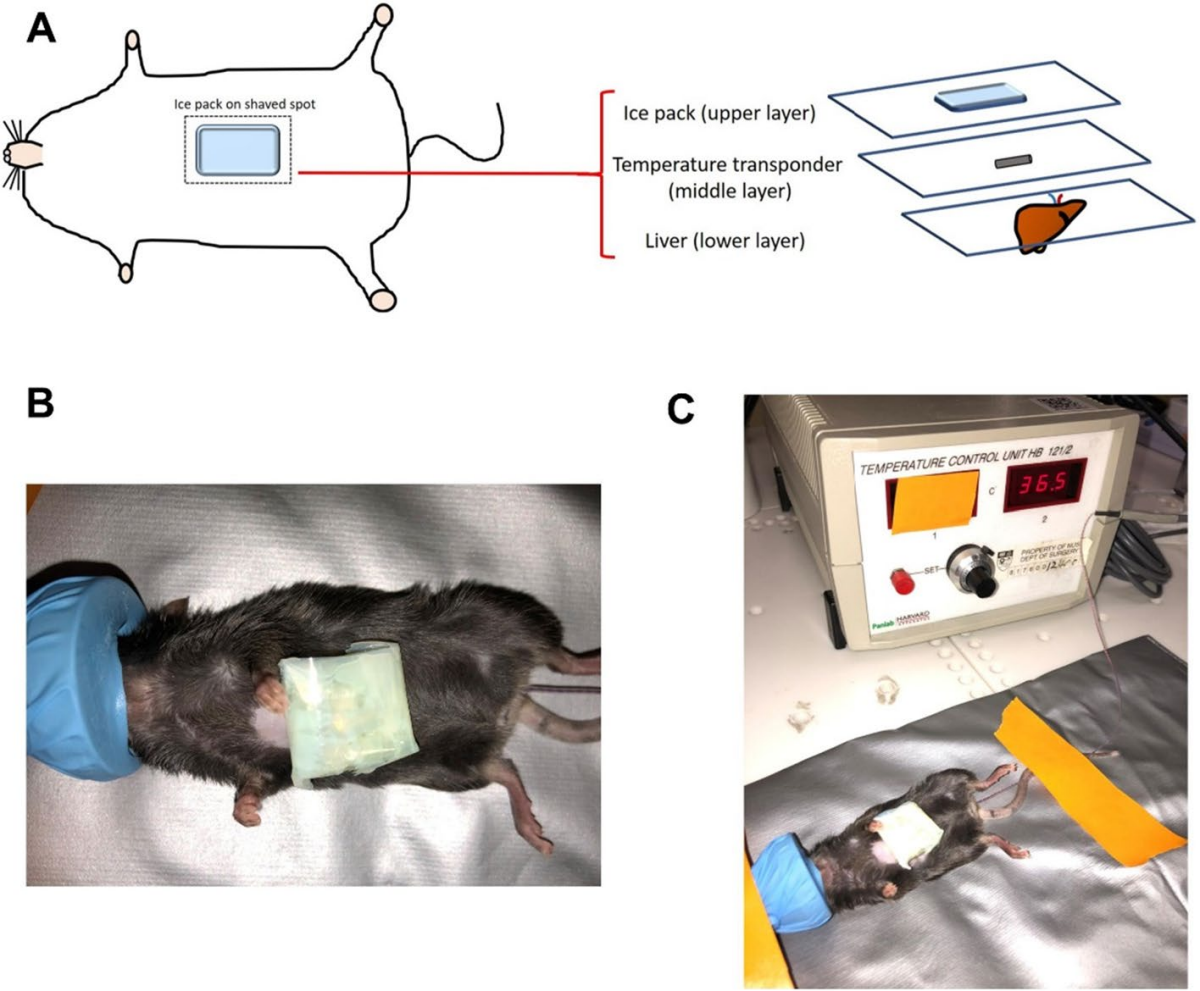
In vivo set-up for targeted cooling of the liver. (**A**) A pictorial representation demonstrates the position of the temperature transponder and the ice pack with respect to the liver. The illustration is not drawn to scale. (**B**) Close-up photograph and (**C**) a wide shot of the actual set-up in mice. Pulsed cooling of the liver is performed with ice pack under isoflurane anesthesia and a rectal probe is used to measure core body temperature of the mice. Throughout the cooling cycle, the mice were placed on a 37 °C heating pad to maintain core body temperature within normal limits.

**Figure 2:**
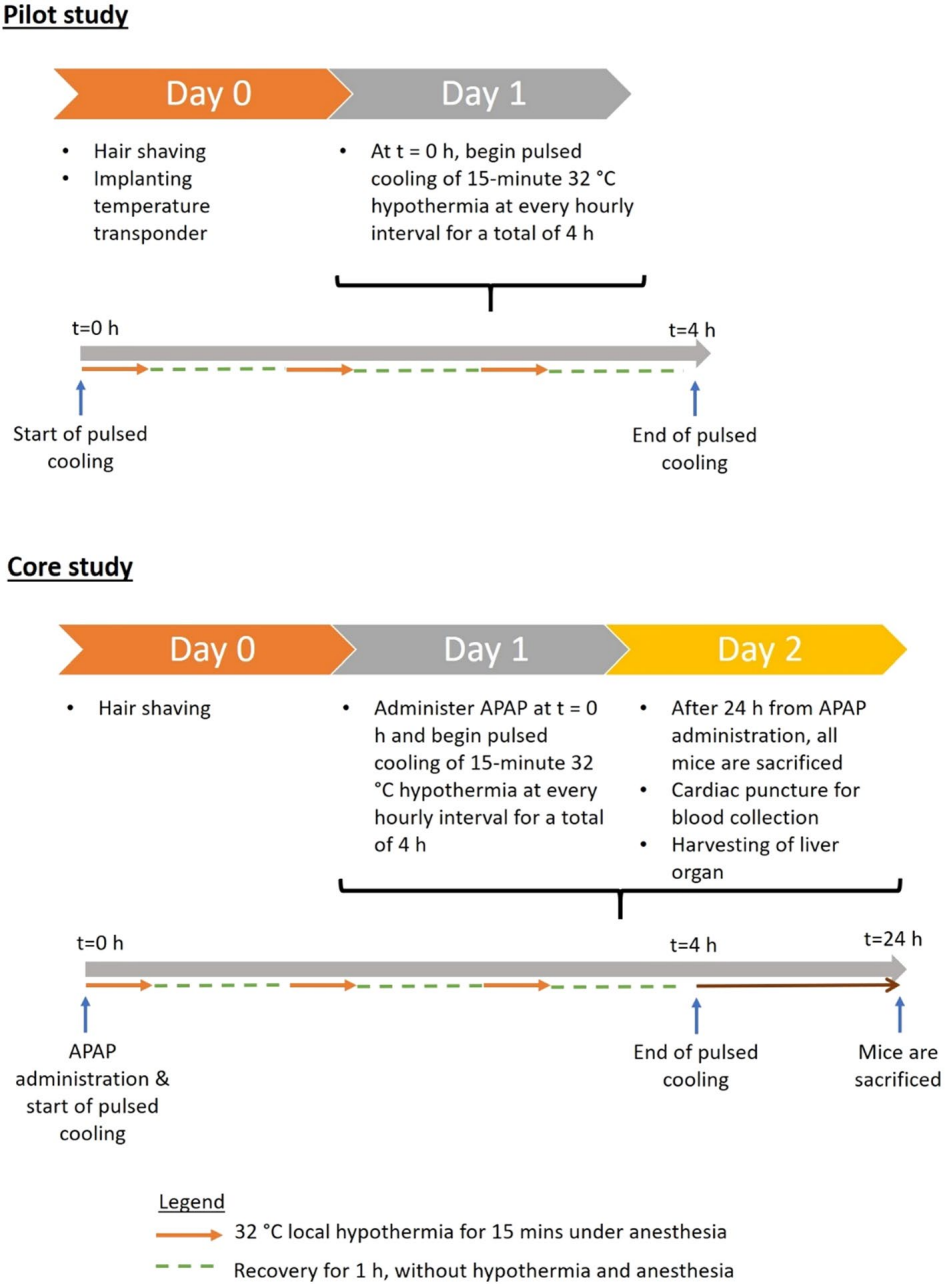
A summary of the in vivo experimental set-up for pilot and core study.

**Figure 3:**
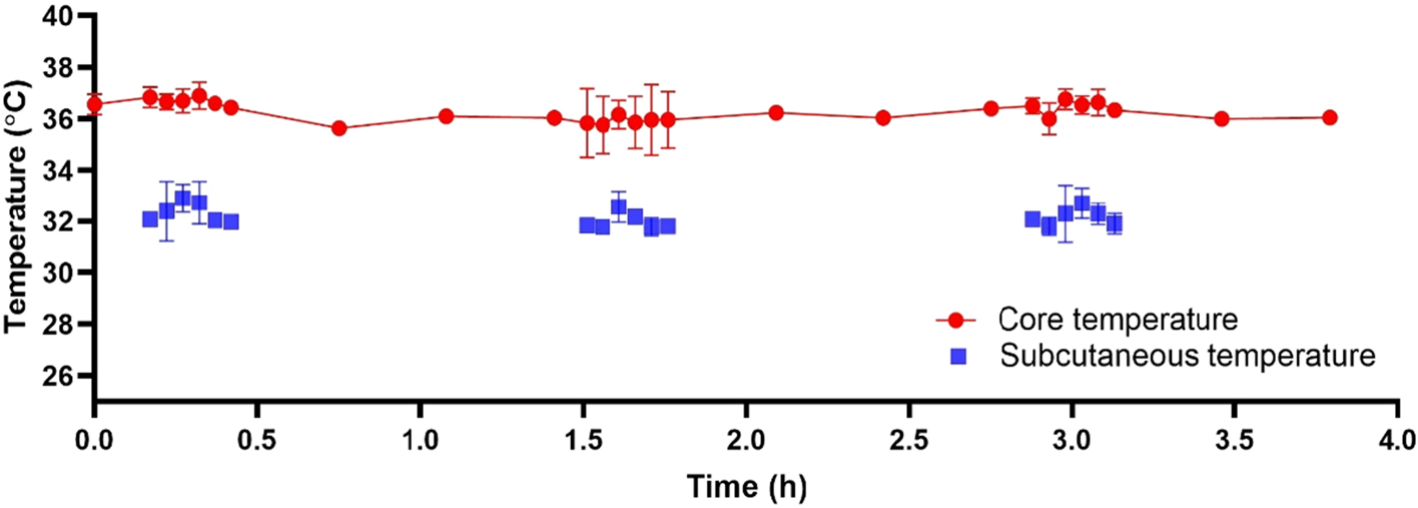
Changes in the core and subcutaneous temperature following pulsed cooling procedure in mice. The core temperature is measured with a rectal probe every 3 min during pulsed cooling, and with an infrared non-contact thermometer gun every 20 min during recovery. Data are presented as mean ± SD (n = 3).

To achieve regional cooling close to the liver, we proposed an empirical pulsed cooling approach comprising short cooling durations with intermittent recovery intervals. Non-invasive cooling was administered periodically by placing an ice pack on the abdomens below the sternum, overlying the anatomical location of the liver (Figs. 1 and 2). The core and subcutaneous body temperature close to the liver was monitored across the four hours of pulsed cooling. A distinct contrast in the two temperatures was measured during each of the cooling cycles-where the liver in proximity achieved cooling within narrow limits of moderate hypothermia (32 ± 0.5 °C) while maintaining a normothermic core body temperature (Fig. 3). This clearly exemplifies the capacity of targeted regional cutaneous cooling to influence local hepatic temperature. As such, it validates the approach for subsequent efficacy study.

### Pulsed cooling could effectively attenuate AILI and improve the overall health status of the subjects

**Figure 4:**
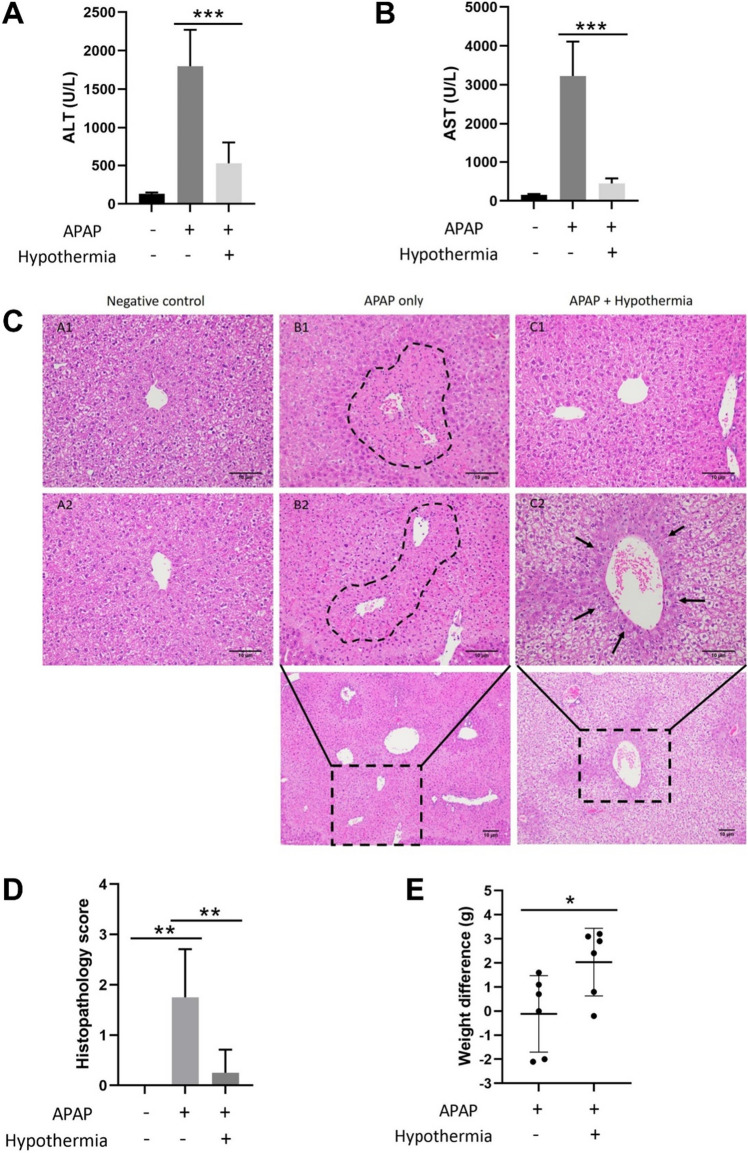
Effect of pulsed cooling on AILI and the overall well-being of mice. Quantitative analysis of (**A**) ALT and (**B**) AST levels were evaluated along with qualitative analysis of liver damage based on (**C**) liver histology using H&E staining. Liver histology in two representative mice in each treatment group is shown. Mice without APAP treatment and without pulsed cooling displayed unremarkable liver histology in both left (A1) and right (A2) liver lobes; mice administered with 300 mg/kg APAP displayed (B1) centrilobular necrosis (left lobe) and (B2) bridging coagulative necrosis between centrilobular zones (right lobe) while mice administered with 300 mg/kg APAP and concomitant pulsed cooling displayed (C1) unremarkable liver histology (left lobe) or (C2) some loss of glycogen (right lobe). (**D**) Histopathological scoring of the degree of liver cell damage was also performed for all mice samples. Finally, mice were weighed before and after 24 h of APAP administration. (**E**) The weight difference was compared between treatment groups, with or without pulsed cooling, as an indicator of the overall well-being of the mice during AILI. Data are presented as mean ± SD (n ≥ 3) where one-way ANOVA was used to make comparison amongst treatment groups and post-hoc tests were subsequently carried out with correction for multiple comparisons using the Sidak’s method. For comparison of weight difference between APAP-treated mice, with or without pulsed cooling, unpaired t-test was used instead. Scale bar = 10 µm. *P < 0.05; **P < 0.01; ***P < 0.001.

To evaluate the effects of pulsed cooling in AILI, liver function tests i.e. alanine transaminase (ALT) and aspartate transaminase (AST) were employed along with the analysis and scoring of the extent of cell damage on liver histology samples. For the former, there was a prominent reduction of ALT and AST levels by more than 70% following pulsed cooling in AILI (Fig. 4A,B); for the latter, centrilobular necrosis was frequently observed in mice with AILI while mice with concomitant pulsed cooling and AILI had almost unremarkable liver histology across both left and right liver lobes, except for occasional reduction of glycogen (Fig. 4C). Overall, pulsed cooling significantly improved the histopathological scoring by at least one grade (Fig. 4D). All these findings collectively demonstrated an effective attenuation of AILI in mice with induced localized hypothermia close to the liver.

Finally, we also assessed the impact of pulsed cooling on the overall well-being of the mice. With intermittent hypothermia, most of the mice achieved a greater weight gain, with statistical significance, after 24 h of APAP treatment (Fig. 4E) compared to the control group that was not treated with induced localized hypothermia. This is evident of a higher feeding rate, which suggests improved overall vitality and activity. These findings suggest that significant deterioration of health and well-being was evaded in the mice that underwent pulsed cooling, in addition to alleviating liver injury.

### RBM3 is the predominant CSP with potential induction during pulsed cooling

**Figure 5:**
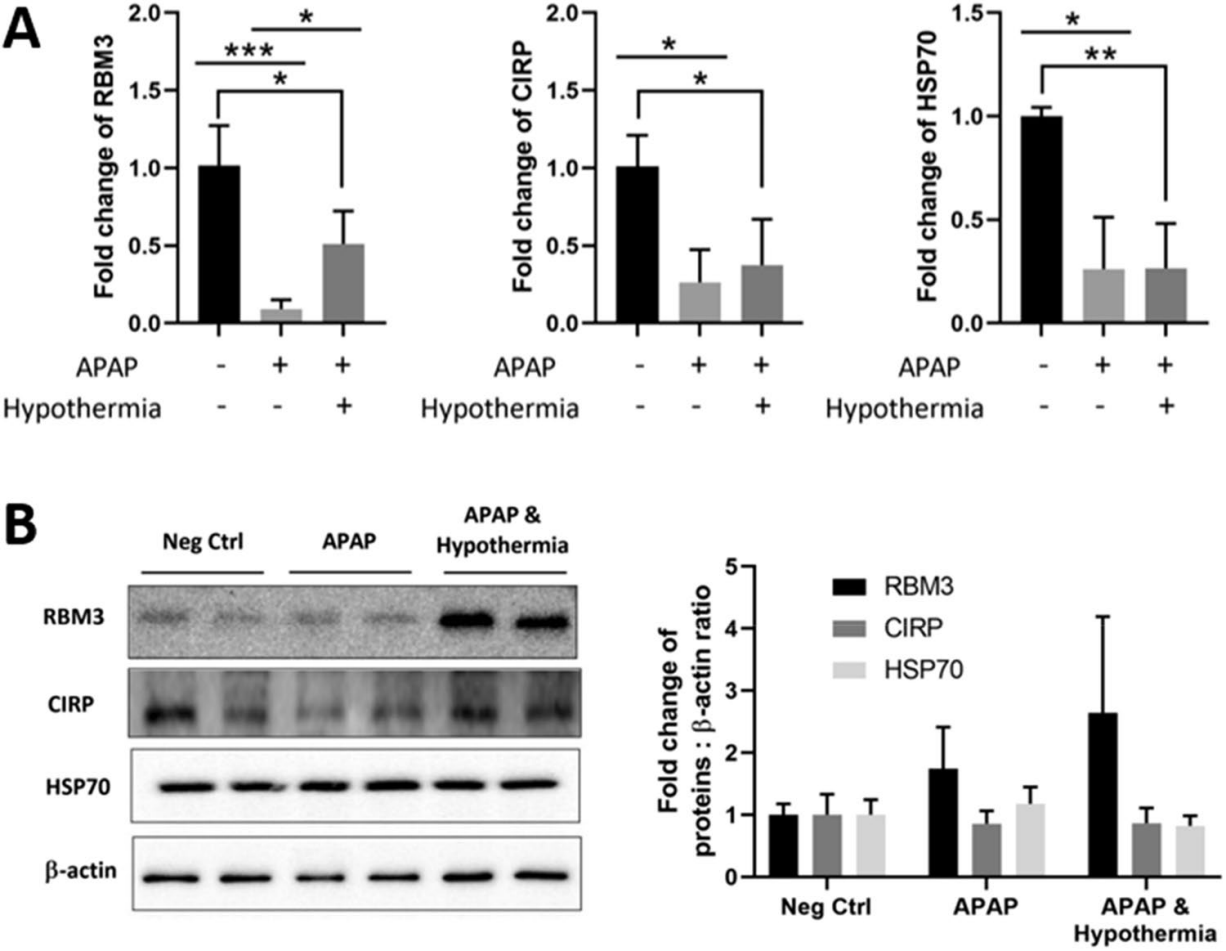
Effect of pulsed cooling on RBM3, CIRP and HSP70 expressions in mice. (**A**) transcript expressions of RBM3, CIRP and HSP70 were explored with real time RT-PCR and β-actin was used as the housekeeping gene. Thereafter, (**B**) western blotting was carried out to investigate the protein expressions of RBM3, CIRP and HSP70. β-actin was used as the housekeeping protein for all western blots. Densitometric analysis was performed with ImageJ and normalized against the negative control i.e. mice without both APAP administration and pulsed cooling. Data are presented as mean ± SD (n ≥ 3) where one-way ANOVA was used to make comparison across different treatment groups and post-hoc tests were subsequently carried out with correction for multiple comparisons using Sidak’s method. *P < 0.05; **P < 0.01; ***P < 0.001.

As intermittent cooling and recovery via pulsed cooling promotes fluctuation of local hepatic temperature, we wish to discern the role of CSP and heat shock protein (HSP) in its underlying hepatoprotective mechanism. Therefore, the transcript and protein expression of well-known CSPs - RNA binding protein motif 3 (RBM3) and cold-inducible RNA binding protein (CIRP) were inquired along with HSP70, an inducible HSP expressed in the liver. Between the two CSPs, RBM3 responds more readily to pulsed cooling than CIRP. An apparent upregulation of RBM3 transcripts was observed, even though its protein induction appears to be masked by mice-to-mice variability (Fig. 5). In contrast, there was unremarkable change in the transcript and protein expression of CIRP (Fig. 5). Similarly, the expression of HSP70 remained unchanged despite a relative rise in hepatic temperature, from moderate hypothermia (32 °C) to normothermia during intermittent recovery phases (Fig. 5). In summary, these observations may imply a predominant cold shock response, potentially involving RBM3, while a heat shock response, encompassing HSP70, would be absent in pulsed cooling.

### Pulsed cooling could advocate concomitant autophagy and mitochondrial biogenesis to alleviate AILI

**Figure 6:**
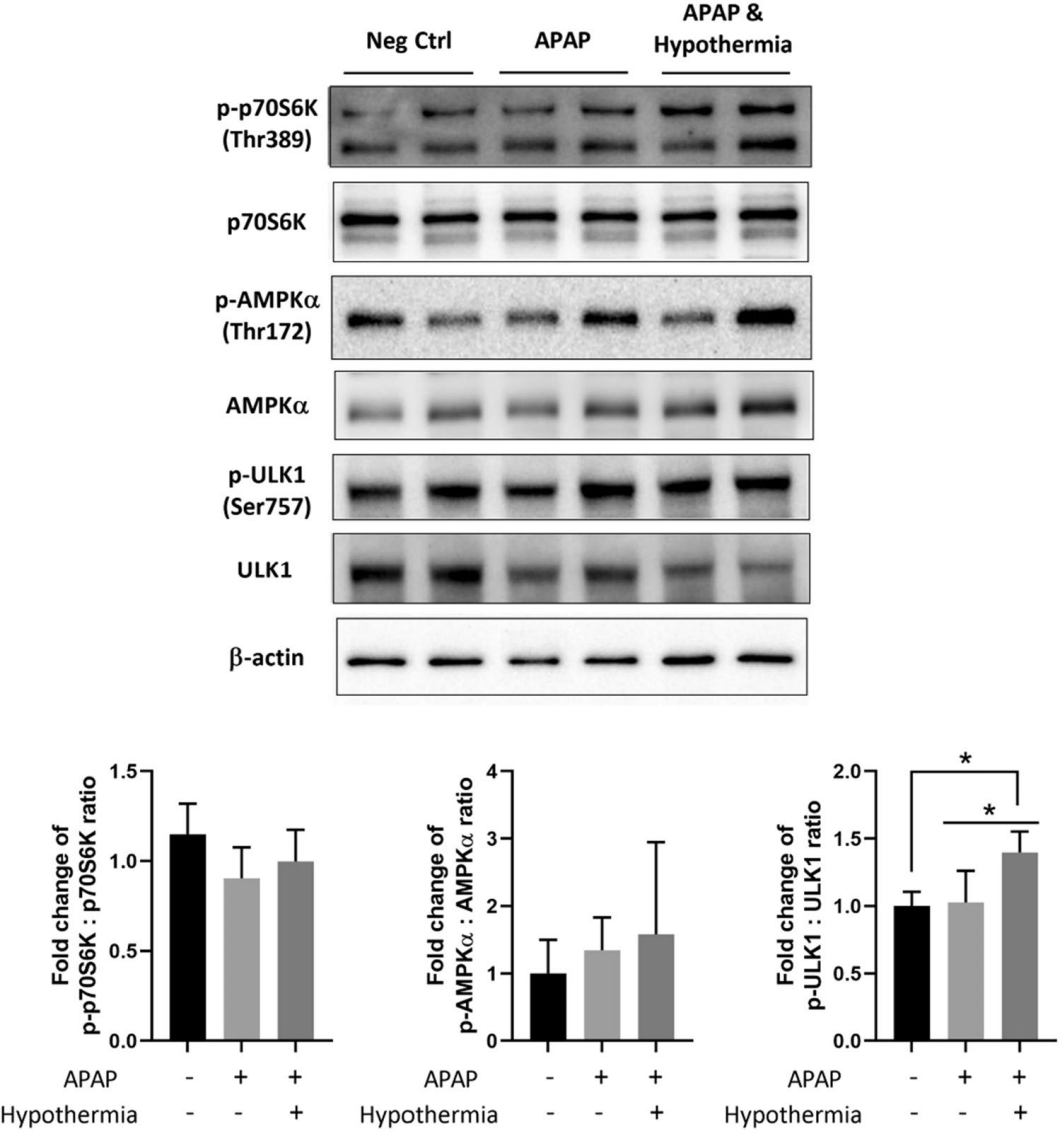
Effect of pulsed cooling on autophagy in mice. Western blotting was carried out to determine the protein expressions of autophagy-related markers including p-p70S6K, p70S6K, p-AMPKα, AMPKα, p-ULK1 and ULK1. β-actin was used as the housekeeping protein for all western blots; densitometric analysis was performed with ImageJ and normalized against the negative control i.e. mice without both APAP administration and pulsed cooling. Data are presented as mean ± SD (n ≥ 3) where one-way ANOVA was used to make comparison across different treatment groups and post-hoc tests were subsequently carried out with correction for multiple comparisons using Sidak’s method. *P < 0.05.

From our past study, moderate hypothermia was shown to render concomitant mitophagy and mitochondrial biogenesis *vitro*^2^. We thus inquired on their existence in a pulsed cooling animal model, to abate mitochondrial dysfunction in AILI. Notably, there was a higher phosphorylation of Unc-51-like autophagy activating kinase 1 (ULK1) with pulsed cooling, a key mediator to facilitate autophagosome-lysosome fusion^8^ and hence, promote autophagy (Fig. 6). To affirm a heightened autophagic phenomenon, we also investigated changes in the activity of well-known upstream regulators of autophagy i.e. mechanistic target of rapamycin complex (mTOR) and AMP-activated protein kinase α (AMPKα). Of which, there was unremarkable difference in the p-p70S6K/p70S6K ratio, which denotes unchanged activity of mTOR (Fig. 6). On the other hand, there was an observed increasing trend of p-AMPKα/AMPKα ratio following hypothermia, which suggests a higher activity of AMPKα, the positive regulator of autophagy (Fig. 6). While its significant activation was seemingly masked by mice-to-mice variability, it appeared to be predominantly responsible for stimulating downstream autophagy, in comparison with mTOR. This illustrates a cold-mediated autophagy occurring with pulsed cooling, with a potential to expel damaged mitochondria and downplay APAP-induced liver damage.

**Figure 7:**
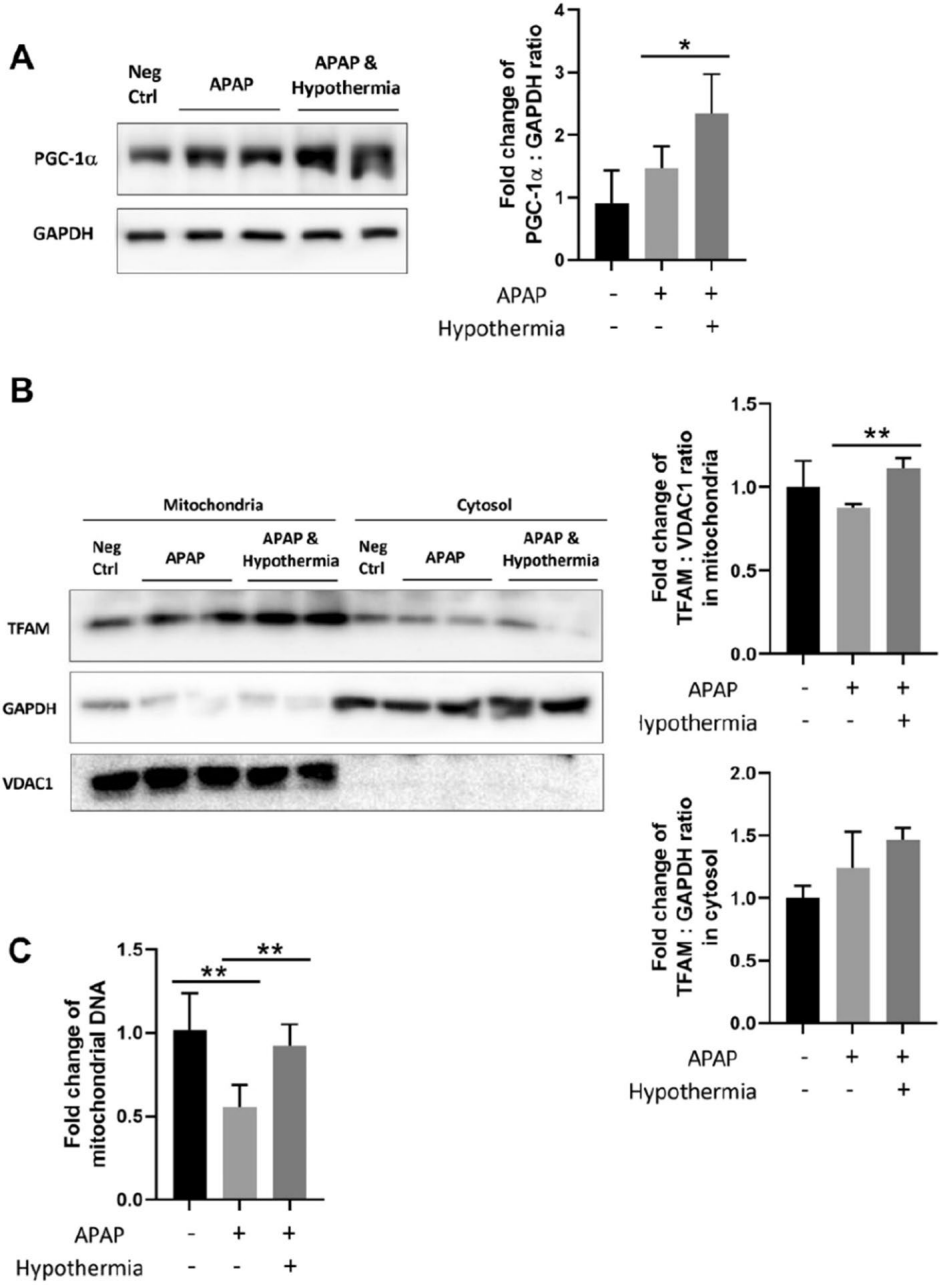
Effect of pulsed cooling on mitochondrial biogenesis in mice. Western blotting was performed to investigate the protein expression of **(A)** PGC-1α in whole cell lysate and (**B**) TFAM in mitochondrial and cytosolic fractions. For whole cell lysate, GAPDH was used as the housekeeping protein; for mitochondrial and cytosolic fractions, VDAC1 and GAPDH was used as the housekeeping proteins respectively. Densitometric analysis was performed for all western blots and normalized against the negative control i.e. mice without both APAP administration and pulsed cooling. (**C**) A relative change in the mitochondrial DNA levels was also determined with real-time RT-PCR. Data are presented as mean ± SD (n ≥ 3) where one-way ANOVA was used to make comparison across different treatment groups and post-hoc tests were subsequently carried out with correction for multiple comparisons using Sidak’s method. *P < 0.05; **P < 0.01.

Concomitantly, mitochondrial biogenesis was shown to occur with pulsed cooling too. With a prominent upregulation of peroxisome proliferator-activated receptor gamma coactivator 1-alpha (PGC-1α), the master regulator of mitochondrial biogenesis, along with heightened levels of transcription factor A, mitochondrial (TFAM) in the mitochondria, they, together, suggest an activation of mitochondrial proliferation (Fig. 7A,B). In fact, a ~ 1.67-fold increase in mitochondrial DNA, upon intermittent hypothermia, was documented too (Fig. 7C). This fosters an increase in mitochondrial mass, and along with autophagy, their concomitant interplay may drive mitochondrial health and elevate hepatoprotection in AILI.

### Pulsed cooling displays antioxidant effects by enhancing GSH conjugation

**Figure 8:**
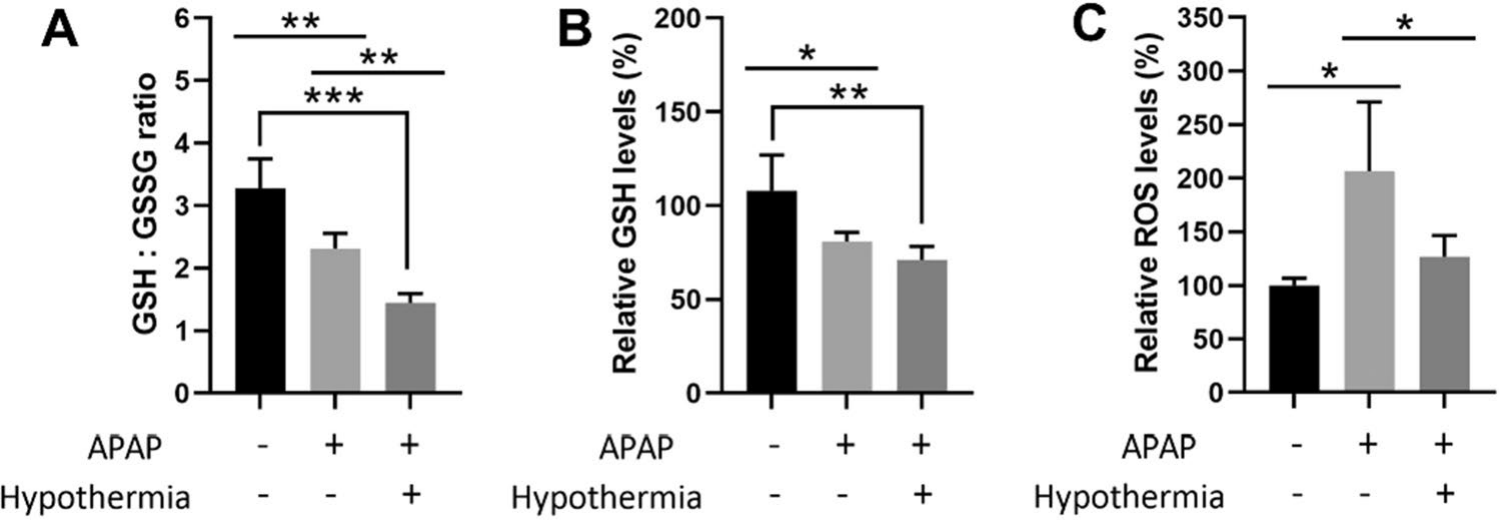
Effect of pulsed cooling on glutathione homeostasis and oxidative stress. (**A**) GSH/GSSG ratio and (**B**) relative GSH levels were determined to examine the flux of GSH recycling and GSH changes respectively. (**C**) The extent of oxidative stress was also inquired by measuring the ROS levels. Data are presented as mean ± SD (n ≥ 3) where one-way ANOVA was used to make comparison across different treatment groups and post-hoc tests were subsequently carried out with correction for multiple comparisons using Sidak’s method. *P < 0.05; **P < 0.01; ***P < 0.001.

Beyond examining the effects of pulsed cooling on mitochondrial damage, we further explored its impact on oxidative stress. With pulsed cooling, it lowered the reduced to oxidized glutathione (GSH:GSSG) ratio, while reduced glutathione (GSH) levels remained low (Fig. 8A,B). Accompanied with a declining trend in reactive oxygen species (ROS) level, these are suggestive of an efficient utilization of GSH for scavenging ROS (Fig. 8C). In other words, pulsed cooling could display desirable antioxidant effects which effectively curtails AILI.

## Discussion

Previous studies have characterized the effects of hypothermia on AILI in vitro^1,2^*.* With this established understanding, we hoped to translate the concept of hypothermic treatment into a clinically actionable therapy-one which achieved hepatoprotection with minimal invasiveness and adverse outcomes. To do so, we applied intermittent, localized cooling close to the liver. This was an empirical procedure in which the liver was subjected to transient hypothermia while maintaining core body temperature. Herein, we have demonstrated, for the first time, the feasibility and efficacy of this non-invasive method in ameliorating AILI in mice.

To perform regional cooling, the use of a small animal model poses some challenges. Mice have a high surface area to volume ratio and high thermal conductance which increases their propensity towards rapid heat loss^9^. As a result, controlled hypothermia at a precise area, without whole body cooling, could be difficult. To address this issue, we placed the mice on a 37 °C heating pad during each cooling cycle to reduce the likelihood of systemic hypothermia. Measurement of regional subcutaneous vs core body temperature during each cooling phase showed a distinct difference (Fig. 3). This established temperature differential has thus allowed for the subsequent core study to evaluate on the efficacy of pulsed cooling in AILI.

To initiate the core study, we standardized the cooling duration to achieve moderate hypothermia at the liver for each cooling cycle i.e. 10 min for the first cycle, 5 min 40 s for the second cycle and 11 min 40 s for the third cycle. These precise cooling timings were derived from the pilot study, based on the mean duration required to cool the mice liver to 32 °C at each cooling cycle. We noted that the second cooling cycle took a shorter time (< 6 min) than the first cycle while the third cycle (> 11 min) took the longest. These findings are in alignment with the reported trends on heat loss upon repeated cold exposure in C57BL/6 J mice^10 ^– that greater heat loss can ensue with repeated cold stimulation, hence a shorter cooling duration may be required for the second cooling phase, compared to the first. Yet, ironically, repeated cooling could also foster cold-induced heat production as a thermoregulatory response in mice. Therefore, this may explain the longer cooling duration of the third cooling cycle. By conducting pulsed cooling in this systematic manner, we hope to streamline the process of cooling and assess its efficacy in a larger study sample.

With the administration of APAP (300 mg/kg) in the core study, acute hepatocellular injury was observed with high levels of ALT and AST, along with prominent centrilobular necrosis on histology (Fig. 4). These liver derangements were however, significantly ameliorated with pulsed cooling (Fig. 4). This localized, intermittent cooling method thus demonstrated hepatoprotection in a similar fashion to conventional continuous cooling^11^. At the same time, with repeated cycles of cooling and recovery, the alleviation of hepatic damage precludes the slowdown of thermodynamics as its primary factor to drive cold-mediated protection. This resonates with our earlier in vitro work which highlighted the role of CSP in driving hepatoprotection, independent of metabolic slowdown^1^.

Amidst the promising findings of reduced liver injury, a loss of glycogen in the hepatocytes was occasionally observed with intermittent cooling (Fig. 4C). This is, however, an expected outcome of cold application, which has been previously reported^12^. Upon cold exposure, glycogen would be employed extensively as the energy substrate while its rate of synthesis would decline. This eventually lead to a depletion of glycogen reserves^13^. By conducting brief periods of cooling with intermittent recovery, we can avoid significant glycogen depletion and preserve the necessary stores known to be crucial for cell survival^14^.

Furthermore, in terms of the overall well-being of mice, pulsed cooling was shown to be associated with better feeding since a higher resultant weight gain was observed after 24 h of AILI (Fig. 4E). This concurred with the findings of Honmore et al. who also reported weight gain in mice following alleviation of AILI^15^. The improved overall well-being of mice thus reinforces the therapeutic potential of pulsed cooling beyond mere hepatoprotection.

Next, we investigated its underlying mechanism and compared against conventional continuous hypothermia with known mechanistic behavior. First, despite frequent temperature cycling between normothermia and hypothermia, HSP70 was not upregulated in pulsed cooling (Fig. 5). Instead, pulsed cooling was more strongly associated with cold-mediated response, where RBM3, rather than CIRP, displayed significant induction (Fig. 5). In a separate transient cooling study, where cells were cooled at 4 °C for two hours followed by 37 °C incubation, the steep temperature elevation from 4–37 °C, failed to promote HSP induction in vitro^16^. This may rationalize the absence of heat-shock response in our pulsed cooling procedure, which comprised of gentler temperature elevations and shorter cooling cycles. By ruling out an interplay of heat-shock mediated protection, it further accentuates the resemblance between pulsed and continuous cooling, where hepatoprotection is driven in a similar manner, involving cold-mediated effects^17,18^. While the inclined induction of RBM3 protein was not consistently observed with pulsed cooling, its augmented transcript expressions raised possibility of a delayed kinetics in mRNA translation for some mice. According to Gottesman et al*.*, translation efficiency can be reduced with cold treatment and an acclimation period of six hours was necessary to recover translational kinetics^19^. Specifically for translation of CSPs, Giuliodori et al*.* reported a preference for them to be translated in cold due to the presence of *cis-*elements in their 5’-UTR of mRNA^20^. Hence, the presence of intermittent recovery in pulsed cooling could have hindered an effective translation of RBM3 mRNA in some mice. As a result, a four-hour empirical pulsed cooling could only manifests cold-mediated effects, independent of CSPs (Figs. 6, 7, 8), unlike conventional hypothermia which exhibits cold-mediated effects through both CSP-dependent and -independent pathways^1,2^. This therefore calls for a need in future study, to further optimize the pulsed cooling protocol to efficiently induce RBM3 proteins.

Second, like conventional hypothermia^2^, there is a concomitant occurrence of autophagy and mitochondrial biogenesis in pulsed cooling (Figs. 6 and 7) and antioxidant effects were prominent too (Fig. 8). Together, they rationalized the hepatoprotective behavior of pulsed cooling in AILI, by downplaying the two key drivers of APAP toxicity-mitochondrial dysfunction and oxidative stress^21^. Interestingly, reduced oxidative stress in pulsed cooling was accompanied with low levels of GSH. This could have stemmed from frequent rewarming in the pulsed cooling procedure. Based on past studies, rewarming from hypothermia may induce oxidative injury due to a sudden mismatch in the oxygen demand and supply at the local site following blood flow restoration^17,22^. Hence, it triggers the GSH to scavenge ROS and lowers GSH:GSSG ratio (Fig. 8). With a global suppression of ROS (Fig. 8C), this underscores a healthy redox environment following the empirical pulsed cooling procedure.

In conclusion, this is the first time a pulsed, localized cooling procedure has been applied to ameliorate DILI, and the results of this empirical in vivo trial have promising implications for future optimization and potential clinical application. The mechanistic similarity with conventional continuous hypothermia may help to build a continuum to define greater therapeutic possibilities of cold physical therapy. On this note, we anticipate further cold applications beyond AILI, for example, for other causes of acute hepatotoxicity and, potentially, for other liver disorders.

## Materials and methods

### Animals

Male C57BL/6NTac mice, of 20–30 g, were purchased from InVivos (Singapore, SG). All animal experimental protocols (R19-1260) have been approved by the National University of Singapore Institutional Animal Care and Use Committee (IACUC). All animal studies were only conducted after three days of acclimatization in the in-house animal housing facility. All methods were carried out in accordance with the relevant guidelines and regulations, and they were reported in accordance with ARRIVE guidelines.

### In vivo pulsed cooling in AILI model

The study began with a pilot phase to establish the feasibility of an empirical pulsed cooling approach, followed by a core investigation of its efficacy in an AILI model in mice.

### Pilot feasibility study

At the skin surface overlying the liver i.e. just below the sternum, the cutaneous hair was shaved over an area of 1.5 cm by 1.5 cm. Thereafter, an IPTT-3000 temperature transponder (BioMedic Data Systems, Seaford, DE), 14 mm by length and 2 mm by diameter, was inserted into the subcutaneous region at the shaved area using a disposable needle assembly according to the manufacturer’s instructions (Fig. 1A). Both the hair shaving and the implantation of temperature transponder were performed under isoflurane (Piramal Critical Care, Bethlehem, PA) anesthesia to avoid potential discomfort on the mice. The mice were allowed to recover overnight before pulsed cooling is performed. On the next day, prior to the start of cooling procedure, at time, t = 0 h, the subcutaneous temperature was measured using the temperature transponder. Due to the proximity of the transponder to the liver, the recorded readings reflected local hepatic temperature. The core body temperature was simultaneously measured, to ensure that the mice were in a normothermic state before further manipulation. The mice were then anesthetized and placed on a heating pad set to 37 °C to maintain the core body temperature within physiological range. Next, an ice pack measuring 1.5 cm by 1.5 cm was placed over the shaved area as depicted in Fig. 1. Subcutaneous temperature was monitored till 32 °C regional hypothermia is achieved, and cooling would be continued for 15 min, during which the ice pack was intermittently removed to prevent excessive drop in temperature below 32 °C. The mice remained anesthetized during local hepatic cooling, while core body temperature was maintained within acceptable physiological limits. After 15 min of local hypothermia at the liver, the anesthesia was withdrawn and the mice were returned to their cages. This entire procedure of anesthetizing and cooling was repeated on an hourly basis for four hours in total. By recording the subcutaneous and core body temperature hourly on three mice, we sought to verify that local cooling at the liver could be achieved, without disruption to the core body temperature during each successive cooling cycle. At the end of the pilot study, the mice were left in the cage for a week to recover from the cooling treatments, before euthanizing them for liver harvesting and blood collection. Euthanasia was performed with carbon dioxide overdose and cervical dislocation. These mice samples were then assigned as the negative controls in the subsequent core efficacy study.

### Core efficacy study

The efficacy of pulsed cooling was evaluated with two groups of mice - one, mice with AILI alone without pulsed cooling; two, mice with AILI that were subjected to concomitant pulsed cooling for four hours. Two mice were randomly assigned to each group and the entire study would be triplicated. The bulk of the procedure remained the same as in the pilot study with two exceptions - first, all mice were injected intraperitoneally with 300 mg/kg of 15 mg/mL APAP (Sigma-Aldrich, St. Louis, MO) dissolved in 0.9% (w/v) sodium chloride solution (B. Braun, Melsungen, DE) just before the start of pulsed cooling. Mice were fasted for 12 h before the administration of APAP; second, a standardized pulsed cooling protocol would be implemented. Regardless of pulsed cooling treatment, mice were anesthetized with isoflurane and placed on heating pad during the designated cooling intervals. The APAP dose was fixed at 300 mg/kg as mice administered with 400 mg/kg (n = 2) failed to survive three rounds of 15-min isoflurane anesthesia. Indeed, long exposure of isoflurane anesthesia has been previously reported to induce liver injury^23^. Therefore, to limit the potential of fatal liver injury, mice were administered with 300 mg/kg APAP. Unlike the pilot study where close temperature monitoring was performed for each mouse to determine the time needed for localized cooling to achieve moderate hypothermia (32 °C), we sought to determine the average time taken to achieve targeted cooling at each cooling cycle based on the pilot study. We then standardized the duration of cooling for each cooling cycle and maintained the mice at moderate hypothermia for 15 min, followed by a one-hour recovery interval between each cooling cycle. Therefore, no temperature transponder was implanted at the site of cooling. Specifically, mice were cooled in a sequential manner for 10 min, 5 min 40 s and 11 min 40 s for the three cooling cycles within a four-hour pulsed cooling procedure. Thereafter, the mice were left in the cage overnight. After 24 h of APAP exposure, the mice were weighed before being euthanized by carbon dioxide overdose and cervical dislocation. After which, cardiac puncture was performed for blood collection and the liver was harvested for histological and biochemical analysis. A summary of the pilot and core study is illustrated in Fig. 2.

### Measurement of subcutaneous and core body temperature

The subcutaneous temperature was measured using an implanted temperature transponder that was inserted into the subcutaneous tissue overlying the liver. The measurement was thus suggestive of the local hepatic temperature. The core body temperature was measured using a rectal thermometer probe (Panlab, Barcelona, ES) during pulsed cooling. By simultaneously measuring both subcutaneous and core body temperature, we aimed to demonstrate a regional hypothermic effect close to the liver, independent of the core body temperature. Both subcutaneous and core body temperature measurements were taken before the start of pulsed cooling, at t = 0 h, and for every three minutes during each of the 15-min cooling cycle; during the hourly recovery period between pulsed cooling, temperature measurements would be performed every 20 min. To protect the mice from physical trauma following frequent insertion of rectal thermometer probe, we used an infrared non-contact thermometer gun (Movel Scientific Instrument, Zhejiang, CN) to measure core body temperature at the rectal region instead, during recovery periods. Its accuracy had been found to be comparable with rectal thermometer probe during hands-on practice in mice.

### Liver function tests

Cardiac puncture was performed on mice after they were euthanized with carbon dioxide overdose and cervical dislocation. Whole blood was collected in serum separator tubes and left at room temperature to allow blood clotting for 30 min. Thereafter, they were transported back to the laboratory where serum samples were isolated by centrifuging whole blood samples at 3500 rpm for ten minutes. For analysis of liver enzymes levels, ALT and AST assay kits (Nanjing Jiancheng Bioengineering Institute, Jiangsu, China) were used according to manufacturer’s instructions. All serum samples were measured in triplicates.

### Histological staining

After euthanasia, the whole liver was harvested from each mouse and part of the liver, including both the left and right liver lobe, was fixed in 10% (v/v) neutral buffered formalin (Sigma-Aldrich, St. Louis, MO). The liver tissue was then processed and embedded in paraffin wax, followed by sectioning and mounting onto polysine ™ slides (Thermo Scientific, Waltham, MA). To visualize the liver histology, liver tissue sections were stained with hematoxylin and eosin (H&E) (Sigma-Aldrich, St. Louis, MO) before visualizing under an Olympus microscope (BX43, Tokyo, JP). For APAP-treated mice, histology was first compared between left and right liver lobes to confirm the distribution of liver injury. In the absence of a marked difference between left and right liver lobes (Supplementary Fig. S1), the comparison between APAP-treated mice, with and without pulsed cooling, was subsequently performed indiscriminately across various liver lobes. Histopathological scoring for the degree of liver cell damage was performed according to the scoring rubrics detailed in Blakzka et al.^24^. Apart from the extent of hepatic congestion and necrosis, the extent of glycogen loss was added as another criterion for grading the severity of APAP-induced liver damage.

### Real-time PCR analysis

The harvested liver tissue was snap-frozen in liquid nitrogen and stored in -80 °C freezer prior to RNA and DNA extraction. To isolate total RNA, approximately 20 mg of mice tissue was first disrupted with Dounce homogenizer, followed by homogenization with QIAshredder (Qiagen, Venlo, NL) and finally extracted using RNeasy mini kit (Qiagen, Venlo, NL). Thereafter, total RNA was quantified with NanoDrop 1000 UV/Vis spectrophotometer (Thermo Scientific, Waltham, MA) and cDNA was synthesized from 1 µg of total RNA using qScript cDNA SuperMix (Quantabio, Beverly, MA) based on manufacturer’s instructions. Real-time PCR analysis was performed using QuantiFast SYBR Green PCR Kit (Qiagen, Beverly, MA) on CFX96 touch real-time PCR detection system (Bio-Rad, Hercules, CA) and the cycling conditions were as follow—the samples were heated at 95 °C for five minutes, followed by 40 cycles of 95 °C for 10 s and 60 °C for 30 s. The relative mRNA expressions were determined based on fold changes calculated using 2^-ΔΔCt^, where normalization was performed against the negative control i.e. mice without both APAP treatment and pulsed cooling. The transcript expressions of RBM3, CIRP and HSP70 were quantified, and β-actin was used as the housekeeping gene. The primer sequences (Integrated DNA Technologies Coralville, IA) are listed in Table 1. All samples were run in triplicates.

**Table 1:** Primer sequences used in real-time PCR.

Gene	Primer sequence (5ʹ → 3ʹ)	Product length (bp)
RNA-binding motif protein 3 (RBM3)-homologous to human RBM3	F: CCTTCACAAACCCAGAGCAT	177
	R: TTCCATATCCCTGGTCTCCA	
Cold-inducible RNA-binding protein (CIRP)-homologous to human CIRP	F: GCGGCAGATCAGAGTTGAC	191
	R: AGCCTCCATAACCCCCACT	
Heat shock protein 1A (HSPA1A)-homologous to human HSP70	F: CAAGATCACCATCACCAACG	237
	R: ATGACCTCCTGGCACTTGTC	
Mitochondrially encoded NADH dehydrogenase 1 (ND1)*-homologous to human ND1	F: CTAGCAGAAACAAACCGGGC	–
	R: CCGGCTGCGTATTCTACGTT	
Hexokinase 2 (HK2)*-homologous to human HK2	F: GCCAGCCTCTCCTGATTTTAGTGT	–
	R: GGGAACACAAAAGACCTCTTCTGG	
Beta-actin (β-actin)	F: TGTTACCAACTGGGACGACA	165
	R: GGGGTGTTGAAGGTCTCAAA	

*Cited from Quiros et al.^26^.

To isolate total DNA, approximately 20 mg of mice tissue were homogenized in the same manner as described above, followed by an extraction using DNeasy blood and tissue kit (Qiagen, Venlo, NL). By conducting real-time PCR analysis as described above, the DNA expression of ND1 would be quantified. For that, hexokinase 2 is used as the housekeeping gene. The primer sequences were listed in Table 1. All samples were run in triplicates.

### Isolation of mitochondrial and cytosolic fraction

To isolate the mitochondrial and cytosolic fraction from liver tissues, approximately 20 mg of frozen liver, was first homogenized with Dounce homogenizer in the mitochondrial isolation buffer (pH 7.5) comprising 50 mM HEPES, 320 mM sucrose, 10 mM potassium chloride, 1.5 mM magnesium chloride, 1 mM EDTA, 1 mM dithiothreitol and protease inhibitor cocktail which included 10 mM sodium fluoride, 100 mM PMSF, 2 M sodium orthovanadate and 2 μg/mL aprotinin. Following on, another round of mechanical homogenization was performed by passing through 25 G needle using a 1 mL syringe to disintegrate cell membranes for the release of cellular fractions. Thereafter, cell debris was removed by centrifuging the cell lysates at 800 g for 10 min. The supernatant was then collected and subjected to further centrifugation at 10,000 g for 20 min. Here, the supernatant was collected as the cytosolic fraction while the resultant pellet forms the mitochondrial fraction. Finally, the pellet was resuspended in cell lysis buffer comprising 10 mM sodium fluoride, 100 mM phenylmethylsulfonyl fluoride (PMSF), 2 mM sodium orthovanadate, 2 μg/mL aprotinin, 1% (v/v) octylphenoxypolyethoxyethanol, 0.5% (w/v) sodium deoxycholate and 0.1% (w/v) sodium dodecyl sulfate (SDS) diluted in PBS. All chemicals stated were obtained from Sigma-Aldrich (St. Louis, MO).

### Western blotting

Approximately 20 mg of the liver tissue was used for protein extraction to characterize various protein expressions with western blotting. To do so, the frozen liver tissue was first homogenized with Dounce homogenizer in the cell lysis buffer as described in earlier section. Thereafter, the proteins extracted in the lysis buffer were isolated through centrifugation at 13,000 rpm for ten minutes at 4 °C and quantified. Western blotting was performed based on our past study^1^. Briefly, 20 μg protein samples were separated via 8% and 15% (v/v) SDS–polyacrylamide gel electrophoresis and transferred onto polyvinylidene fluoride (PVDF) membranes (Bio-Rad, Hercules, CA) for all proteins, with the only exception of CIRP using nitrocellulose membrane (Thermo Scientific, Waltham, MA). All PVDF membranes were blocked with 5% (w/v) BSA in tris-buffered saline containing 0.1% (v/v) Tween-20 (TBS-T) while nitrocellulose membrane was blocked with 5% (w/v) skim milk in TBS-T. Thereafter, all membranes were incubated overnight at 4 °C in primary antibodies diluted in 1:1000, except anti-β-actin antibody which was diluted in 1:10,000. Next, all membranes were washed with TBS-T before incubating with horseradish peroxidase-conjugated secondary antibodies (Cell Signaling, Danvers, MA), at 1:10,000 dilution, for an hour at room temperature. The protein bands were then visualized with chemiluminescence image analyzer (G:BOX Chemi XX6, Syngene, Cambridge, UK) using western lightning plus-ECL reagent (PerkinElmer, Waltham, MA). All protein bands were quantified with ImageJ software (National Institutes of Health, Maryland, US) and normalized against the housekeeping protein, β-actin or GAPDH; for phosphorylated proteins i.e. p-p70S6K, p-AMPKα and p-ULK1, they were normalized against their corresponding total protein levels; for TFAM in the specific mitochondrial and cytosolic fractions, it was normalized against the housekeeping proteins, VDAC1 and GAPDH, respectively. All chemicals stated were obtained from Sigma-Aldrich (St. Louis, MO).

### Measurement of hepatic GSH content and GSH/GSSG ratio

20 mg of frozen liver tissue was homogenized with a Dounce homogenizer in the cell lysis buffer as described in earlier section. The relative hepatic GSH content in mice samples were determined with glutathione cell-based detection kit (Cayman Chemical, Ann Arbor, MI) according to manufacturer’s instructions. By using monochlorobimane as the GSH substrate, it reacts with GSH and the resultant fluorescence intensity was measured at an excitation wavelength of 380 nm and at an emission wavelength of 480 nm on a microplate reader. Relative changes, by percentage, in the GSH content were determined by normalizing against the negative control i.e. mice without both APAP administration and pulsed cooling. All samples were run in triplicates.

To further examine the extent of reduced (GSH) and oxidized glutathione (GSSG) levels, GSH/GSSG ratio detection assay kit (Abcam, Cambridge, UK) was used according to manufacturer’s instructions. With the use of thiol green fluorophore, the fluorescence intensity representing the total glutathione and reduced GSH were measured at an excitation wavelength of 490 nm and at an emission wavelength of 520 nm on a microplate reader. Following on, the level of GSSG was calculated and GSH/GSSG ratio was determined. All samples were run in triplicates.

### Measurement of ROS levels

The measurement of ROS levels in the liver tissue was performed as previously described^25^. Briefly, 20 mg of frozen liver tissue was homogenized with a Dounce homogenizer in 200 µL of 40 mM Tris–HCl buffer (pH 7.4) (Bio Basic, Markham, ON). Next, 100 µL of the homogenate was mixed with 1 mL of Tris–HCl buffer, containing 10 µM of DCFDA (Sigma-Aldrich, St. Louis, MO). The mixture was then incubated, with orbital shaking, at 37 °C for 40 min. The resultant fluorescence intensity was measured at an excitation wavelength of 485 nm and an emission wavelength of 535 nm on a microplate reader. Relative changes in the ROS levels were determined by normalizing against the negative control i.e. mice without both APAP treatment and pulsed cooling. All samples were run in triplicates.

### Statistical analysis

Data was expressed as mean ± SD of three biological replicates in the pilot study, and as mean ± SD of six biological replicates in the core study. Statistical analysis was carried out using GraphPad Prism for Windows (version 7.00) (GraphPad Software, La Jolla, CA). One-way ANOVA was used to compare across all groups i.e. mice without APAP treatment and pulsed cooling, mice with APAP treatment alone and mice with both APAP treatment and pulsed cooling. Post-hoc tests were subsequently carried out with correction for multiple comparisons using the Sidak’s method. For comparison of weight difference between APAP-treated mice, with or without pulsed cooling, two-tailed unpaired student’s t-test, of equal variance, was performed. Differences between groups were considered statistically significant for *P* < 0.05.

## Supplementary Information

Supplementary Information.
