# Supplementary material for: In vivo demonstration of a novel non-invasive model for inducing localized hypothermia to ameliorate hepatotoxicity
Source: Sci Rep. 2021 Sep 20;11:18620. doi: 10.1038/s41598-021-98078-6 (PMC8452685; doi:10.1038/s41598-021-98078-6)
Supplement: Supplementary file 1 — Supplementary Information. [file 41598_2021_98078_MOESM1_ESM.docx]

***In vivo* demonstration of a novel non-invasive model for inducing localized hypothermia to ameliorate hepatotoxicity**

Yeong Lan Tan ^1,2^, Min En Nga^3^ and Han Kiat Ho ^1,2,^*

^1^ Department of Pharmacy, Faculty of Science, National University of Singapore, Singapore 117543, Singapore

^2^ NUS Graduate School for Integrative Sciences & Engineering, Centre for Life Sciences, National University of Singapore, Singapore 119077, Singapore

^3^ Department of Pathology, National University Hospital, Singapore 119074, Singapore

***** Correspondence: phahohk@nus.edu.sg

**Supplementary Figure**


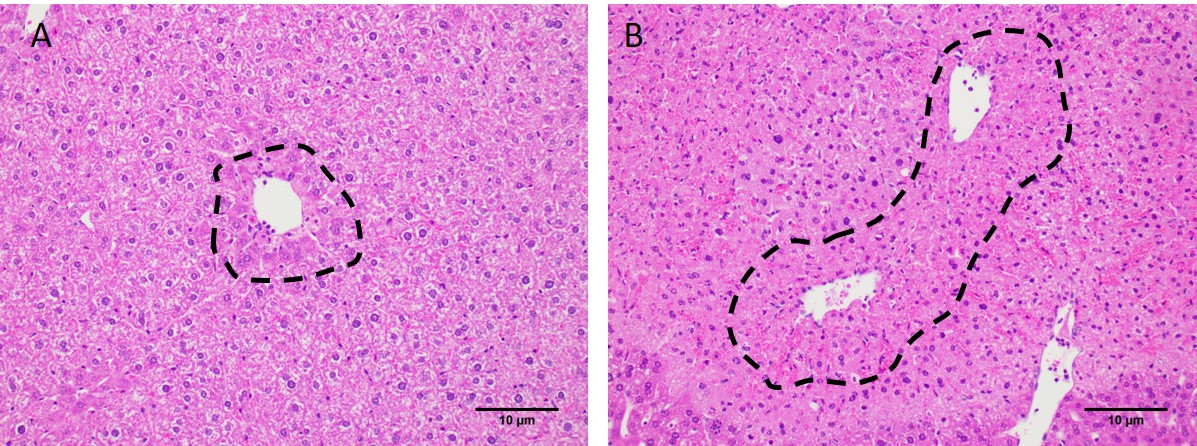


**Supplementary Figure S1. Extent of AILI in left and right liver lobe of APAP-treated mice.** Liver histology of (A) left and (B) right liver lobe of APAP-treated mice is shown. Mice administered with 300 mg/kg APAP incurred centrilobular necrosis in bilateral liver lobes. Scale bar = 10 µm.


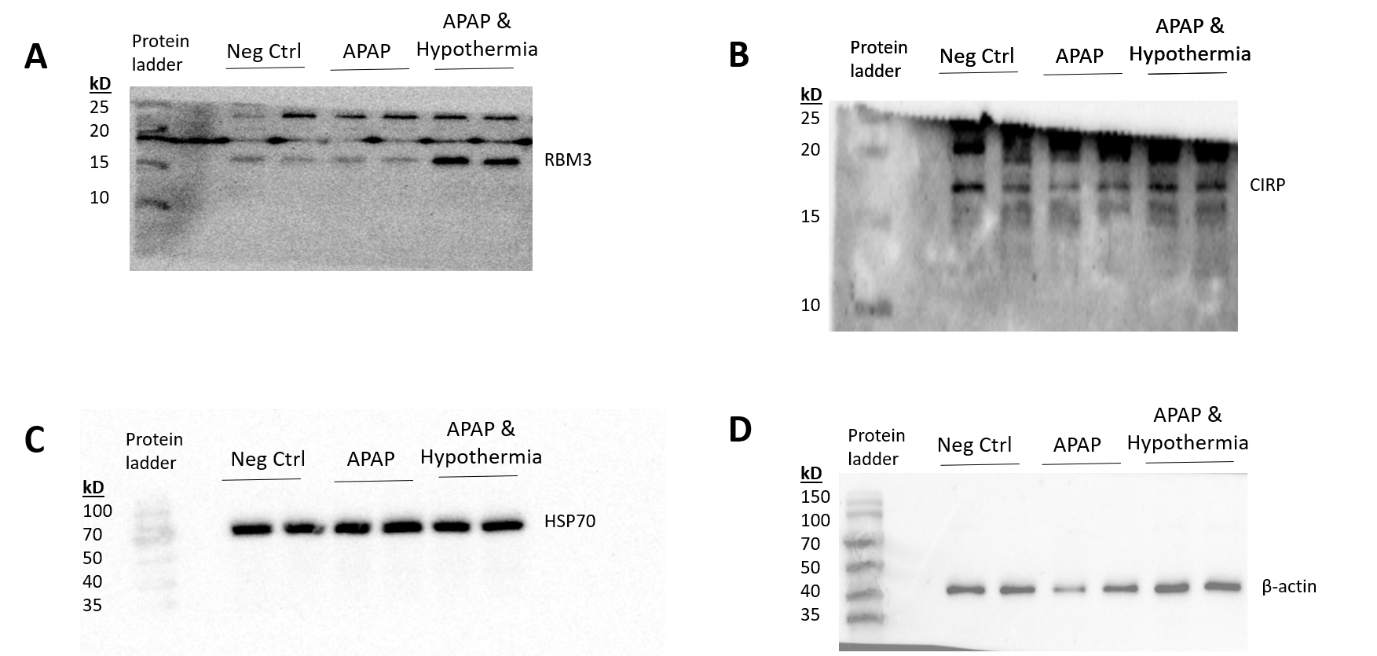


**Supplementary Figure S2.** **Effect of pulsed cooling on RBM3, CIRP and HSP70 expressions in mice.** Full length western blots of (A) RBM3, (B) CIRP and (C) HSP70 protein expressions are shown. (D) β-actin was used as the housekeeping protein for all western blots.

**
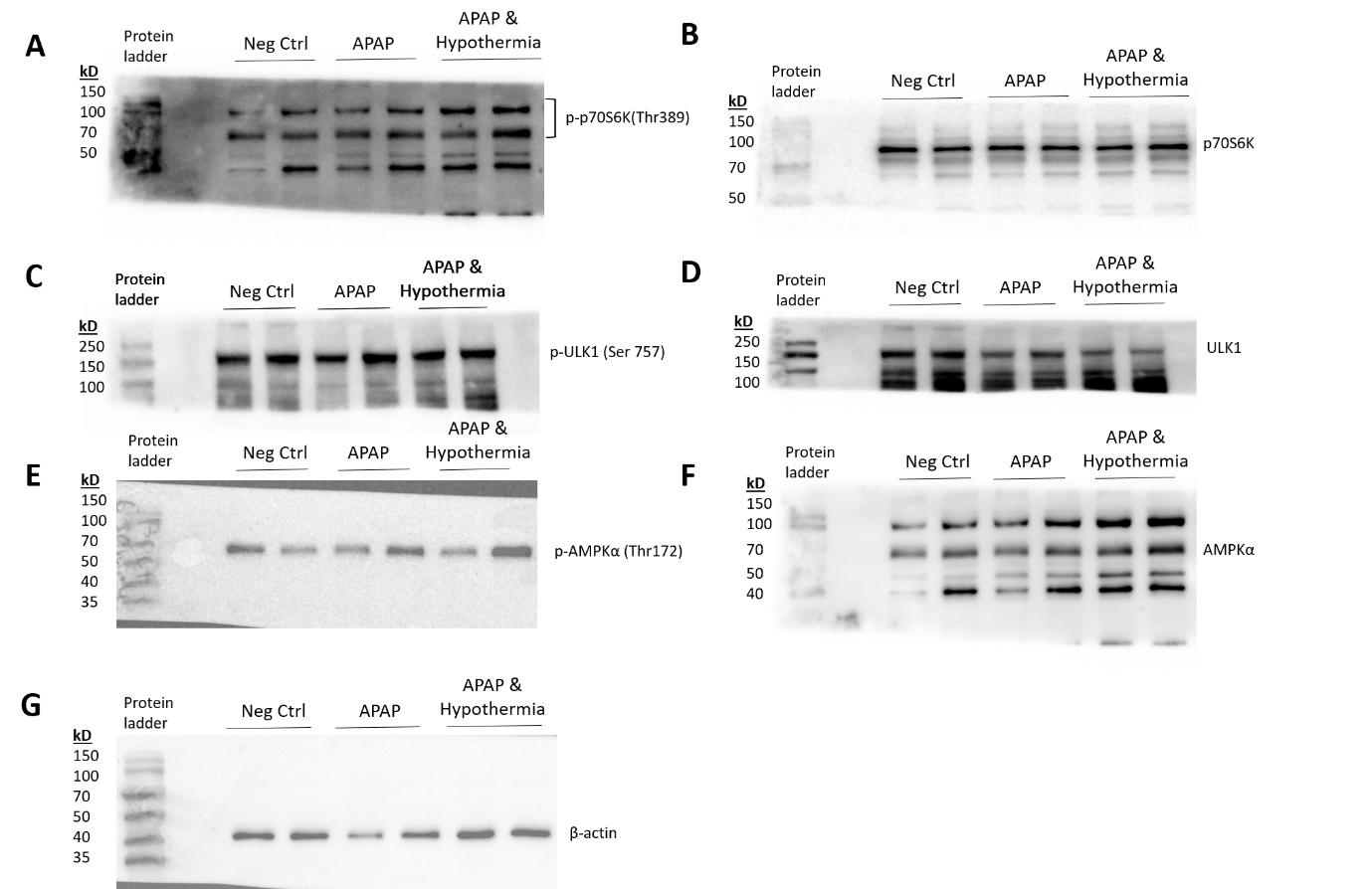
**

**Supplementary Figure S3.** **Effect of pulsed cooling on autophagy in mice.** Full length western blots of (A) p-p70S6K, (B) p70S6K, (C) p-AMPKα, (D) AMPKα, (E) p-ULK1 and (F) ULK1 protein expressions are shown. (G) β-actin was used as the housekeeping protein for all western blots.

**
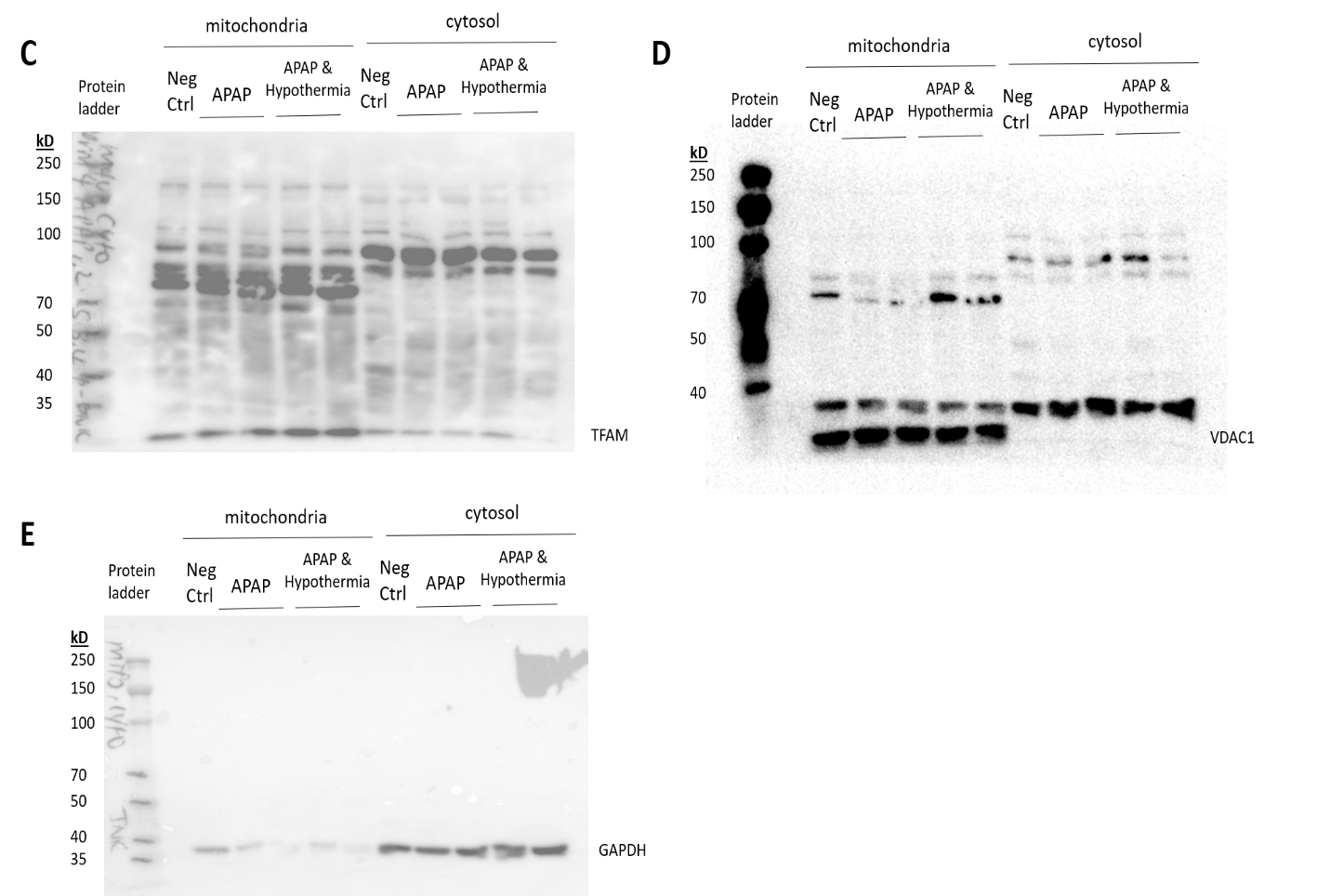
**
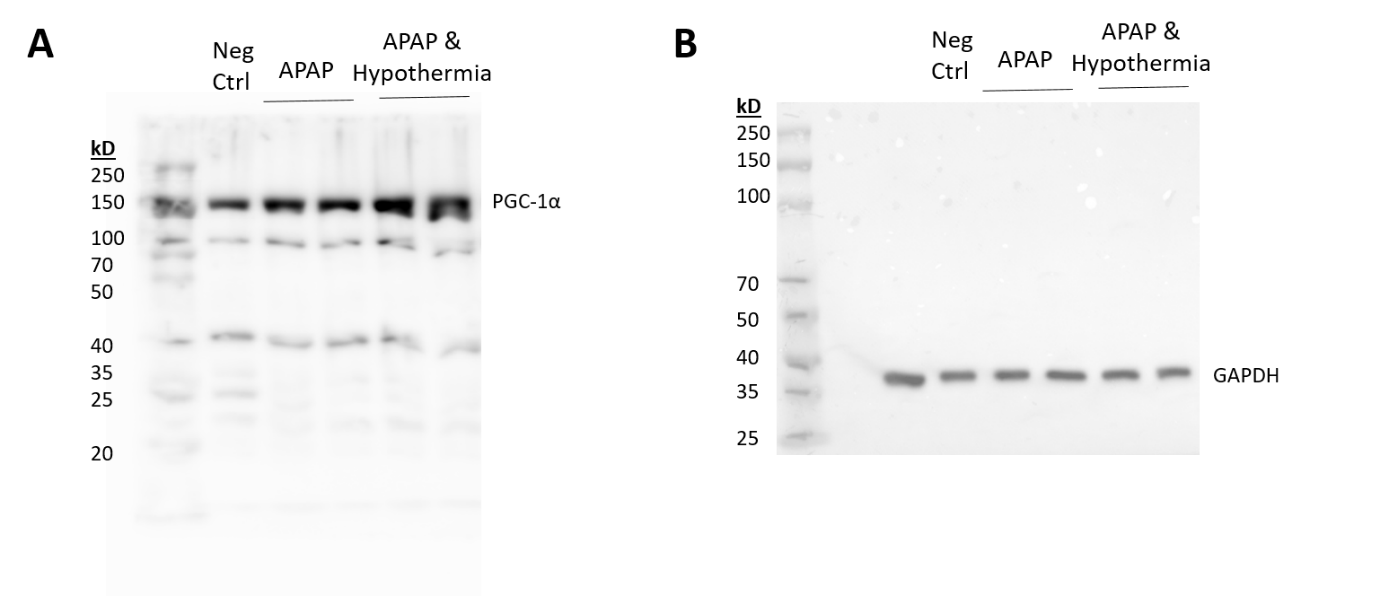


**Supplementary Figure S4.** **Effect of pulsed cooling on mitochondrial biogenesis in mice.** Full length western blot of (A) PGC-1α in whole cell lysate is shown. (B) GAPDH is used as the housekeeping protein in whole cell lysate. This is followed by full length western blots of (C) TFAM protein expression in mitochondrial and cytosolic fractions. (D) VDAC1 and (E) GAPDH was used as the housekeeping proteins for mitochondrial and cytosolic fractions respectively.
